# Wie häufig ist die persistierende Tubenventilationsstörung nach erfolgter Gaumenspaltenoperation wirklich? Einsatz neuester Diagnostik

**DOI:** 10.1007/s00106-022-01147-z

**Published:** 2022-03-22

**Authors:** Nora M. Weiss, Franziska Bennöhr, Jan-Hendrik Lenz, Robert Mlynski, Stefanie Rettschlag

**Affiliations:** 1 Klinik für Hals-Nasen-Ohrenheilkunde, Kopf- und Halschirurgie „Otto Körner“, Universitätsmedizin Rostock, Doberaner Str. 137–139, 18057 Rostock, Deutschland; 2 Klinik und Poliklinik für Mund‑, Kiefer- und Plastische Gesichtschirurgie, Klinik und Polikliniken für Zahn‑, Mund- und Kieferheilkunde, Universitätsmedizin Rostock, Rostock, Deutschland

**Keywords:** ETS‑7, ETDQ‑7, Diagnosewerkzeug, Chronisch obstruktive Tube, Eustachi-Röhre, ETS‑7, ETDQ‑7, Diagnostic tool, Chronic obstructive Eustachian tube

## Abstract

**Fragestellung:**

Bei Patienten mit einer Lippen-Kiefer-Gaumen-Spalte (LKGS) wird eine hohe Inzidenz von chronisch-obstruktiven Tubenventilationsstörungen auch nach chirurgischem Spaltverschluss angenommen. Folglich ist eine erhöhte Rate von Trommelfellretraktionen oder Cholesteatomen zu erwarten. Ziel dieser Studie war es, mit den aktuellen Methoden die Prävalenz chronisch-obstruktiven Tubenventilationsstörung bei erwachsenen Patienten nach behandelter LKGS zu untersuchen.

**Methoden:**

Es wurden erwachsene Patienten mit LKGS nach chirurgischem Spaltverschluss in der Kindheit eingeschlossen und mit einer Kontrollgruppe verglichen. Eine Nachuntersuchung erfolgte mittels Tympanometrie, Tubenmanometrie, ETDQ‑7 (Eustachian Tube Dysfunction Questionnaire) und dem Eustachian Tube Score‑7 (ETS-7).

**Ergebnisse:**

Insgesamt 16 Erwachsene nach LKGS-Operation und 40 gesunde Probanden wurden eingeschlossen. Signifikante Unterschiede wurden beim medianen ETS-7-Score (*p* < 0,0001) gefunden, nicht aber beim medianen ETDQ-7-Score (*p* = 0,09). Nur 2 der 32 untersuchten Ohren (6 %) wiesen sowohl einen pathologischen ETS‑7 als auch ETDQ‑7 auf. Bei 5 (31 %) LKGS-Patienten ergab sich gemäß ETS‑7 Anhalt für eine Beeinträchtigung der Tubenfunktion. Kein Patient hatte einen Untersuchungsbefund, der eine weitere Behandlung notwendig machte.

**Schlussfolgerung:**

Durch die Kombination von Diagnoseinstrumenten für chronisch-obstruktive Tubenventilationsstörungen wurde eine geringere Rate an persistierenden chronisch-obstruktiven Tubenventilationsstörungen bei Erwachsenen nach LKGS-Operation gefunden als bisher berichtet. Eine erfolgreiche chirurgische Behandlung der LKGS scheint nach langfristiger Nachbeobachtung zu einer physiologischen Funktion der Tuba auditiva zu führen.

**Zusatzmaterial online:**

Die Online-Version dieses Beitrags (10.1007/s00106-022-01147-z) enthält zwei Fragebögen (Eustachian Tube Score‑7, ETS‑7, und Eustachian Tube Dysfunction Questionnaire‑7, ETDQ-7).

## Abstract (en)

**Objective:**

In patients with cleft palate, a high incidence of chronic obstructive Eustachian tube dysfunction (ETD) is assumed after surgery. Consequently, an increased rate of retracted eardrum or cholesteatoma is expected. Even though no common standard for investigating ETD is available, the development of objective tests has increased during the past 10 years. This study aimed to investigate the incidence of persisting chronic obstructive ETD in adult patients with cleft palate surgically treated in early childhood by using a combination of diagnostic tools.

**Methods:**

Adult participants with cleft palate repair in early childhood were invited for a follow-up examination and compared to a control group. Examination included tympanometry, tubomanometry, the Eustachian Tube Dysfunction Questionnaire (ETDQ-7), and the Eustachian Tube Score 7 (ETS-7).

**Results:**

A total of 16 adult patients were re-examined after cleft palate surgery and compared to a control group of 40 healthy individuals. Significant differences were found in the median ETS‑7 (*p *< 0.0001) score but not in the median ETDQ‑7 (*p* = 0.09) score. Only 2 of the 32 investigated ears (6%) had pathologic scores in ETS‑7 and ETDQ‑7 (*p* = 0.09), whereas 5 (31%) cleft palate patients showed symptoms of chronic obstructive ETD according to ETS‑7. No patient had examination findings indicating a requirement for further treatment.

**Conclusion:**

By combining diagnostic tools for ETD, a lower rate of persisting obstructive ETD in adults after cleft palate surgery was found than previously reported. Successful surgical treatment of cleft palate seems to lead to physiological Eustachian tube function in long-term follow up.

Kinder mit Lippen-Kiefer-Gaumen-Spalte (LKGS) leiden häufig an rezidivierenden Mittelohrergüssen aufgrund einer chronisch-obstruktiven Ventilationsstörung der Tuba auditiva (TA). Die Prävalenz von Lippenspalten liegt bei 3,28 pro 10.000 und die der Lippen-Kiefer-Gaumen-Spalte bei 6,64 pro 10.000 Neugeborene [1]. LKGS entstehen durch einen unvollständigen Gewebeverschluss im sekundären Gaumen zwischen der 7. und 12. Schwangerschaftswoche. In jedem Fall steht bei der individuellen Behandlung ein interdisziplinärer, zentrumsbezogener Teamansatz im Vordergrund: Die chirurgische Einlage einer Paukendrainage ist eine häufig indizierte Begleittherapie und wird oft im Rahmen einer primären LKGS-Operation durchgeführt. Es wird angenommen, dass Patienten mit LKGS unter einer persistierenden chronisch-obstruktiven TA leiden und ein höheres Risiko haben, im Jugendalter eine chronische adhäsive Otitis media oder ein Cholesteatom zu entwickeln [2]. Der Einfluss der Funktion der TA auf die Pathophysiologie der chronischen Mittelohrerkrankung wird jedoch kontrovers diskutiert. Außerdem wird die Untersuchung der Funktion der TA durch das Fehlen eines Goldstandards für die Diagnose und Therapie der chronisch-obstruktiven TA erschwert [3, 4]. Die TA ist verantwortlich für den Druckausgleich im Mittelohr, den Schallschutz und die Drainage des Mittelohrs [5, 6]. Typische Symptome der chronisch-obstruktiven TA sind ein Druckgefühl im Ohr, das sich bei Änderungen des atmosphärischen Drucks verstärkt, und die Unfähigkeit, den Druckausgleich manuell mit dem Valsalva-Manöver durchzuführen. Weitere Beschwerden können gedämpftes Hören, rezidivierende Paukenergüsse oder Cholesteatome sein [7]. Diese Beschwerden können die Lebensqualität des Patienten stark beeinträchtigen [8]. Bis heute bestehen die üblichen Diagnoseinstrumente aus der mikroskopischen Inspektion des Trommelfells, dem Valsalva- und Toynbee-Manöver und der Tympanometrie [9]. Diese Tests sind umfassend und leicht zugänglich, setzen aber ein intaktes Trommelfell voraus und erzielen nicht zuverlässig reproduzierbare und quantifizierbare Ergebnisse. Es konnte gezeigt werden, dass die Fragebögen allein nicht zwischen Patienten mit klaffender Tube, obstruktiver Tube und Tinnitus unterscheiden und damit nur indirekte Rückschlüsse auf die Tubenfunktion erlauben [10]. Experimentelle Ansätze wie eine Visualisierung der TA in Magnetresonanztomographie oder Computertomographie [11] sowie Videoendoskopie und Sonotubometrie haben sich in der Routine nicht etabliert [12].

In der letzten Dekade wurden mehrere Versuche unternommen, die Diagnose einer chronisch-obstruktiven TA zu verbessern. Die Tubenmanometrie (TMM) wurde erstmals im Jahr 2001 als semi-objektive Methode zur Erfassung der TA-Funktion beschrieben [13]. Der Eustachian Tube Score‑7 (ETS-7) ist ein Instrument zur Beurteilung der chronisch-obstruktiven TA, das anamnestische Informationen und Untersuchungsergebnisse kombiniert. Er gilt als wertvolles Follow-up-Instrument, um den Erfolg der TA-Therapie zu untersuchen [14]. Außerdem wurde der Eustachian Tube Dysfunction Questionnaire‑7 (ETDQ-7) als Fragebogen zur Dysfunktion der TA entwickelt, um subjektive Beschwerden einer TA-Dysfunktion zu bewerten [15]. Es konnte jedoch gezeigt werden, dass der ETDQ‑7 nicht sicher dazu in der Lage ist, suffizient zwischen klaffender und obstruktiver Dysfunktion der TA zu unterscheiden [16]. Da es bisher keine Goldstandard zur Diagnostik von chronisch-obstruktiven Tubenfunktionsstörungen gibt, wird eine Kombination mehrerer Messungen empfohlen und in bisherigen Studien erfolgreich angewendet [10, 14, 17, 18].

Ziel dieser Studie war, die langfristige Beeinträchtigung der Funktion der TA bei Erwachsenen mit abgeschlossenem körperlichen Wachstum nach LKGS-Operationen zu evaluieren.

## Methoden

### Studiendesign und Patientenauswahl

In dieser Querschnitts-Kohortenstudie wurden erwachsene Patienten eines universitären Spalt-Zentrums mit einer Vorgeschichte einer chirurgischen Behandlung von Spalten des weichen Gaumens oder des harten und weichen Gaumens eingeschlossen. Untersucht wurden ausschließlich kaukasische Patienten+innen mit nichtsyndromalen, kompletten einseitigen (LAHS bzw. SHAL) oder/und beidseitigen Lippen-Kiefer-Gaumen-Segelspalten (LAHSHAL). Alle Patienten wurden nach dem standardisierten Schema des Rostocker Spaltzentrums von der Geburt bis zum 18. Lebensjahr behandelt: Der einseitige oder beidseitige Lippenverschluss erfolgte im Alter von 6 Monaten mit der Wellenschnittplastik nach Pfeifer [19]. In gleicher Narkose erfolgten die binokulare Mikroskopie, Parazentese und Einlage von Paukenröhrchen beidseits. Im Alter von 9–12 Monaten wurde der operative Verschluss des Weichgaumens im Rahmen eines zweizeitigen Therapiekonzepts mit der intravelaren Veloplastik nach Kriens [20] durchgeführt.

Der Verschluss des harten Gaumens erfolgte operativ mit vier Lebensjahren in Abhängigkeit von der Breite der Restspalten mit der Brückenlappentechnik [21] oder der Stiellappentechnik [22]. Im Rahmen der Lippenplastik mit 6 Lebensmonaten erfolgte bei keinem der untersuchten Patienten eine primäre anteriore Septumplastik. Ebenso wurde eine Septorhinoplastik nach Wachstumsabschluss nicht standardisiert durchgeführt.

Die Patienten unterzogen sich zwischen Januar 2017 und Dezember 2017 in der Nachuntersuchung einer kompletten HNO-Untersuchung einschließlich Ohrmikroskopie, Tympanometrie, Audiometrie, Tubenmanometrie und Erhebung von ETDQ‑7 (Supplement 1) und ETS‑7 (Supplement 2). Patienten mit Autoimmunerkrankungen, Verdacht auf Tumoren der Kopf-Hals-Region und Patienten nach Strahlentherapie des Kopfes und Halses wurden ausgeschlossen. Die Kontrollgruppe bestand aus gesunden Probanden ohne Vorgeschichte einer Ohrerkrankung oder Ohroperation. Für die Studie liegt ein positives Votum der lokalen Ethikkommission vor (Registriernummer A2016-0150).

### Art der LKGS

Die verschiedenen Arten von LKGS wurden nach dem LAHSHAL-Dokumentationssystem klassifiziert (L = Lippe; A = Alveole; H = harter Gaumen; S = weicher Gaumen) [23]. Da isolierte Lippen- und Gaumensegelspalten ausgeschlossen wurden, wurden Patienten mit kompletten Spaltbildungen des harten und weichen Gaumens mit beidseitiger Lippenspalte (LAHSHAL) und kompletten Spaltbildungen des harten und weichen Gaumens mit einseitiger Lippenspalte (LAHS oder SHAL) analysiert.

### Audiometrische Beurteilung

Alle audiometrischen Messungen wurden mit kalibrierten Geräten in einem schallisolierten Raum (DIN EN ISO 8253) durchgeführt. Die Tympanometrie wurde bei 226 Hz durchgeführt. Die Tympanometrie galt als normal, wenn die Impedanzkurve ein Compliance-Maximum bei < −100 mm H_2_O aufwies (Typ A). Ein pathologisches Tympanogramm lag vor, wenn die Impedanzkurve flach war (Typ B) oder ein Compliance-Maximum unter > −100 mm H_2_O zeigte (Typ C) [24].

### Funktion der Tuba auditiva

#### ETDQ-7

Der EDTQ‑7 wurde 2012 entwickelt und wurde in mehrere Sprachen übersetzt und validiert, um Patienten mit chronisch-obstruktiver TA zu untersuchen. Er besteht aus 7 Fragen, die sich auf Symptome der Ohren beziehen, wie z. B. ein Völlegefühl im Ohr und gedämpftes Hören. Die Antworten werden auf einer numerischen Antwortskala von 1 bis 7 angegeben. Hohe Punktzahlen korrelieren mit einer größeren subjektiven Beeinträchtigung. Der Cut-off-Punkt für die Diagnose einer chronisch-obstruktiven TA liegt bei ≥ 14,5 bei 100 % Sensitivität und 100 % Spezifität. Er wurde als krankheitsspezifisches Instrument für Patienten mit chronisch-obstruktiver TA entwickelt und kann auch nach chirurgischen Eingriffen eingesetzt werden [14].

#### ETS-7

Der ETS‑7 ist ein Instrument zur Untersuchung der chronisch-obstruktiven TA, das anamnestische Informationen und Untersuchungsergebnisse kombiniert. Er umfasst die Bewertung objektiver Messungen wie Tympanometrie und Tubenmanometrie sowie subjektive Beschwerden wie Ohrdruck und subjektives positives Valsalva-Manöver. Der ETS‑7 reicht von 0 bis 14 Punkten. Werte ≤ 7 werden als pathologisch definiert und weisen auf eine chronisch-obstruktive TA hin [13].

#### Tubenmanometrie

Die TMM wurde erstmals von Estève [13] beschrieben und ermöglicht die Beurteilung der TA-Funktion durch Applikation definierter Drücke (30, 40 und 50 mbar) durch die Nase in den Epipharyngealraum. Der Schluckmechanismus löst die Öffnung des knorpeligen Teils der TA aus und dichtet gleichzeitig den Epipharyngealraum vorübergehend ab. Bei normaler TA-Funktion wird der entstehende Druck auf das Mittelohr übertragen. Eine Druckrezeptorsonde, die den äußeren Gehörgang abdichtet, registriert Druckänderungen, die durch Bewegungen des Trommelfells übertragen werden. Der R‑Wert als Hauptergebnisparameter bezeichnet die Latenzzeit zwischen Druckapplikation im Epipharyngealraum und der Messung der Druckänderung im äußeren Gehörgang. R‑Werte ≤ 1 zeigen eine zeitgerechte Tubenöffnung an, R‑Werte > 1 zeigen eine verzögerte Öffnung der TA an. Nicht definierbare R‑Werte sind mit keiner nachweisbaren Öffnung des TA verbunden. In der klinischen Anwendung ist die TMM ein zuverlässiges Instrument, um die Diagnose einer chronisch-obstruktiven TA zu unterstützen [17].

### Statistische Auswertung

Alle statistischen Tests wurden vor der Datenerfassung ausgewählt. Statistische Analysen wurden mit Microsoft Excel und Prism (Version 8, Fa. GraphPad Software, La Jolla, CA, USA) durchgeführt. Das Signifikanzniveau wurde auf *p* < 0,05 festgelegt. Die Normalverteilung wurde durch visuelle Inspektion von Quantil-Quantil-Diagrammen getestet. Wenn nicht anders angegeben, werden die Daten als Median mit Interquartilsbereichen (IQR) oder als absolute Zahlen mit Prozentsätzen dargestellt. Unterschiede zwischen 2 Gruppen wurden mithilfe des Mann-Whitney-U-Tests berechnet. Um Mediane von mehr als zwei Gruppen zu vergleichen, wurde der Kruskal-Wallis-Test verwendet. Unterschiede zwischen Medianen wurden mithilfe der Hodge-Lehmann-Differenz des Medians angegeben.

## Ergebnisse

Insgesamt 16 Patienten (4 Frauen [25 %], 12 [75 %] Männer) mit einem Durchschnittsalter von 25,3 Jahren [SD 4,1] erklärten sich zur Teilnahme an der Studie bereit. Folglich wurde eine Anzahl von 32 Ohren analysiert. Die Tab. 1 zeigt die Patientencharakteristika. Bei 11 (69 %) Patienten wurde eine Anamnese für eine oder mehrere chirurgische Paukenröhrchen-Insertionen und bei 12 (75 %) Patienten eine Anamnese für eine rezidivierende Otitis media erhoben. Nur bei einem Ohr (3 %) wurde eine Cholesteatom-Operation (Tympanoplastik Typ III) in der Anamnese angegeben. In allen Fällen war der Epipharyngealraum frei von Weichteilschwellungen, und der Tubenwulst war bei allen Patienten frei einsehbar. Bei 13 (81 %) Patienten wurde eine (angeborene) Septumdeviation zur Nichtspaltseite festgestellt [25], und 2 (13 %) Patienten hatten Hinweise auf Verwachsungen in der Nasenhöhle.

**Table 1:** 

		Gaumenspalte *n* = 16 Patienten *n* = 32 Ohren	Kontrolle *n* = 40 Patienten *n* = 80 Ohren
*Alter – Jahre*	–	25,3 (SD 4,1)	23,8 (SD 3,7)
*Geschlecht (m/w)*	–	12/4	12/28
*Untersuchung – n (%)*	Freier Epipharynx	16 (100)	40 (100)
	Adhäsivprozess	0 (0,0)	0 (0,0)
	Retraktion Trommelfell	11 (34,3)	0 (0,0)
	Positiver Valsalva	27 (84,3)	79 (98,8)
	Vernarbungen Nasenhöhle	2 (12.5)	0 (0,0)
*Anamnese – n Patienten (%)*	Zurückliegende Cholesteatom-Op.	1 (3,0)	0 (0,0)
	Paukendrainageninsertion	11 (68,8)	0 (0,0)
*Tubenfunktion – Median (IQR)*	ETS‑7	10 (7–12)	12 (11–14)
	ETDQ‑7	10 (7–11)	8 (7–10)
*Tympanogramm – Typ, n (%)*	A	26 (81,3)	79 (98,8)
	B	4 (12,5)	1 (2,5)
	C	2 (6,2)	0 (0,0)
*Art der Gaumenspalte – n (%)*	LASHAL	7 (43,8)	0 (0,0)
	LAHS	5 (31,2)	0 (0,0)
	SHAL	4 (25,0)	0 (0,0)

*LAHS* bzw. *SHAL* einseitige Lippen-Kiefer-Gaumen-Segelspalten, *LAHSHAL* beidseitige Lippen-Kiefer-Gaumen-Segelspalten, *ETS-7* Eustachian Tube Score‑7 (ETS-7), *ETDQ-7*  Eustachian Tube Dysfunction Questionnaire‑7

Die Ohrmikroskopie zeigte in 21 (65,6 %) Ohren unauffällige Befunde. Bei 11 (34,3 %) Ohren zeigte das Trommelfell leichte Retraktionen ohne Verwachsungen oder ein Cholesteatom. Der objektive Valsalva-Test war in 5 (16 %) Ohren negativ. Die restlichen 27 (84 %) Ohren zeigten einen positiven Valsalva-Test. Vier (13 %) Ohren zeigten ein Tympanogramm vom Typ B, 26 (81 %) Ohren zeigten ein Tympanogramm vom Typ A, und 2 (6 %) Ohren zeigten ein Tympanogramm vom Typ C. Der mediane ETS-7-Score betrug 10 (IQR 7–12), was auf normale Werte hinweist. Neun (21,1 %) Ohren (6 betroffene Patienten, 3 unilateral, 3 bilateral) zeigten einen pathologischen ETS-7-Score ≤ 7. Der ETDQ‑7 zeigte einen Medianwert von 10 (IQR 7‑11), was auf normale Werte hindeutet. Bei 4 (12,5 %) Ohren (2 bilateral betroffene Patienten) zeigte der ETDQ‑7 Werte, die mit einer chronisch-obstruktiven TA vereinbar waren. Bei Patienten mit einseitiger Spalte wurden die ipsi- bzw. kontralateralen Ohren miteinander verglichen. Es ließen sich keine Unterschiede in den ETS-7- (*p* = 0,62) und EDTQ-7- (*p* > 0,99) Scores zwischen den beiden Ohren nachweisen. Zusammenfassend wiesen 5 (31 %) Patienten gemäß ETS‑7 eine chronisch-obstruktive TA auf. Bei 4 von 32 Ohren (13 %) wies der ETDQ‑7 Werte für eine chronisch-obstruktive TA auf. Bei nur zwei (6 %) Ohren zeigten sich pathologische Werte sowohl für den ETS‑7 als auch für den ETDQ‑7. Nur eines (3,1 %) dieser Ohren zeigte eine leichte Trommelfellretraktion, das andere zeigte einen normalen Ohrbefund. Kein Patient hatte Untersuchungsbefunde, die eine weitere Behandlung erforderlich machten.

Die Kontrollgruppe bestand aus 40 Teilnehmern (28 [70 %] Frauen, 12 [30 %] Männer) mit einem Durchschnittsalter von 23,8 Jahren [SD 3,7]. Der objektive Valsalva-Test war in 54 (67,5 %) der untersuchten Ohren eindeutig positiv, in 25 (31,3 %) Ohren war er schwach positiv und in einem (1,2 %) Ohr war er negativ. Ein (1,25 %) Ohr zeigte ein Tympanogramm Typ B, die restlichen 79 (98,8 %) Ohren zeigten ein Tympanogramm Typ A. Der mediane ETS-7-Score lag bei 12 (IQR 11–14), was auf normale Werte hinweist. Kein Teilnehmer zeigte pathologische ETS-7-Werte. Der mediane ETDQ-7-Score lag bei 8 (IQR 7–10) und zeigte normale Werte an.

**Figure 1:**
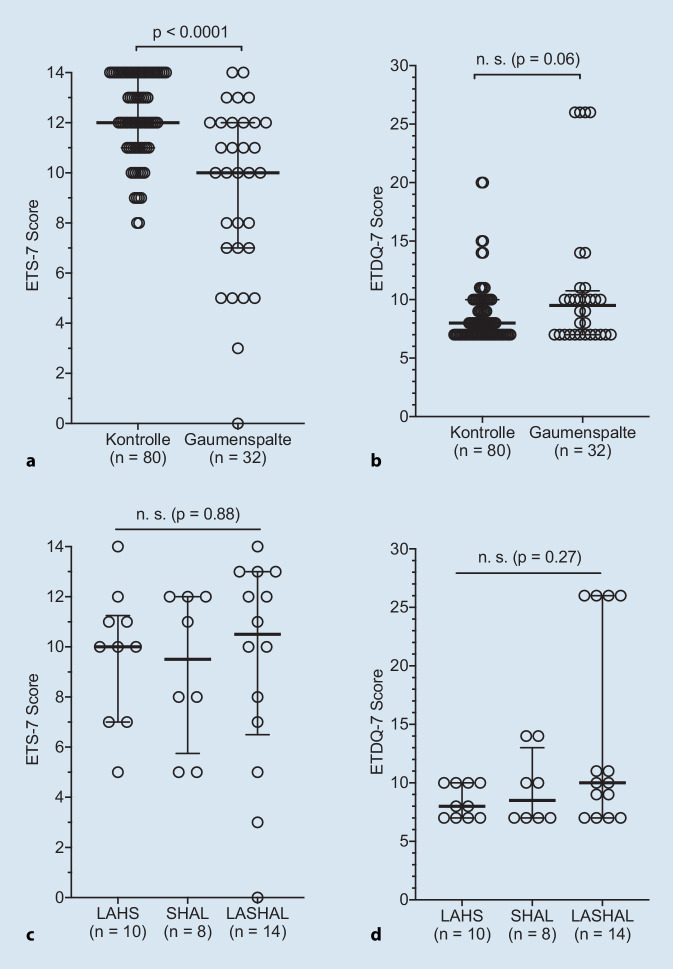

Der Vergleich der Ergebnisse zwischen der LKGS-Patienten und der Kontrollgruppe zeigte signifikante Unterschiede im medianen ETS-7-Score (Hodge-Lehmann-Differenz des Medians: 2, *p* < 0,0001; Abb. 1a), jedoch nicht im medianen ETDQ-7-Score (Hodge-Lehmann-Differenz des Medians: 0, *p* = 0,09; Abb. 1b).

Es wurden keine signifikanten Unterschiede für den ETS‑7 (Abb. 1c) und den ETDQ‑7 (Abb. 1d) zwischen den verschiedenen LKGS-Typen gefunden.

## Diskussion

Entgegen der bisherigen Annahme, dass eine hohe Rate an chronisch-obstruktiver TA bei Patienten mit LKGS vorliegt [26], deuten die Ergebnisse dieser Studie darauf hin, dass nur wenige Erwachsene nach Abschluss der chirurgischen Behandlung der LKGS und nach Abschluss des Längenwachstums an einer persistierenden chronisch-obstruktiven TA leiden. Die Prävalenz der chronischen Tubenventilationsstörung wird mit etwa 1–5 % der erwachsenen Bevölkerung angegeben [27, 28]. Bei Kindern bis zu einem Alter von 10 Jahren entwickeln etwa 40 % mindestens eine Episode einer Dysfunktion der TA [26]. Im hier untersuchten Kollektiv von Erwachsenen nach einer LKGS-Operation lag der Anteil von chronisch-obstruktiver TA nur bei 6 %, eine latente chronisch-obstruktive TA lag bei 31 % vor. Keiner der Patienten hatte einen Paukenerguss oder eine behandlungsbedürftige Mittelohrerkrankung.

Es ist bekannt, dass Kinder mit LKGS ein erhöhtes Risiko für akute und chronische seröse oder muzinöse Paukenergüsse haben [29, 30]. Doyle et al. verwendeten den Forced Response Test (FRT) und den Inflation-Deflation-Test bei 41 Kindern und Jugendlichen mit LKGS, um die Funktion der TA zu beurteilen. Eine chronisch-obstruktive TA mit der Unfähigkeit, Valsalva- und Toynbee-Manöver durchzuführen, wurde bei 73 % festgestellt. Eine Schwäche des M. tensor veli palatini an der Öffnung der TA wurde als Erklärung für diese Ergebnisse angesehen [31]. Alper et al. verglichen diese Daten mit einem eigenen Kollektiv von 31 Ohren in einer Langzeit-Follow-up-Studie. Sie verwendeten ebenfalls den FRT und berichteten von einer Rate von 38 % persistierender chronisch-obstruktiver TA nach Therapie der LKGS [32]. Nach Odoi führt eine weiche Gaumenspalte zu einer veränderten Zugrichtung des M. tensor veli palatini und in der Folge zu einer beidseitig beeinträchtigten Tubenöffnung [33]. Matsune et al. diskutieren eine abnorme Insertion des M. tensor veli palatini in die lateralen Anteile der TA als Ursache für die chronisch-obstruktive TA [34, 35]. Weiterhin werden die Länge der TA und der Winkel zwischen der Lamina lateralis und medialis des Tubenknorpels als verantwortliche Komponenten der chronisch-obstruktiven TA bei Patienten mit LKGS angegeben [36, 37]. Smith et al. berichten von 44,4 % mit bilateraler und 28,4 % mit unilateraler chronisch-obstruktiver TA bei Patienten 6 Jahre nach LKGS-Operation. Allerdings lag das mittlere Patientenalter in dieser Studie nur bei 7 Jahren, und das Längenwachstum und insbesondere das Wachstum des Schädels waren noch nicht abgeschlossen [38]. Ähnlich wie die Kohorte dieser Studie berichteten Gudziol et al. über eine Gruppe von 40 erwachsenen Patienten mit einem mittleren Alter von 19,9 Jahren nach einer LKGS-Operation. Bei 8 (25 %) Patienten lag beidseitig ein pathologisches Tympanogramm vor, und bei weiteren 2 Patienten war das Tympanogramm nur auf der Spaltseite pathologisch. Ein beidseitig normales Tympanogramm wurde bei 22 (68,8 %) Patienten gefunden. Bei 31 % wurde eine chronisch-obstruktive TA diagnostiziert [26]. Damit lag der Anteil mit einem pathologischen Tympanogramm höher als in unserer Studie. Die Bewertung der TA-Funktion erfolgte jedoch nur anhand der Tympanometrie. Da das Tympanogramm allerdings lediglich eine Momentaufnahme liefert, ist es allein für die Diagnose der chronisch-obstruktiven TA nicht geeignet. Wäre die Diagnose in der hier vorgestellten Kohorte dieser Studie allein auf der Grundlage des Tympanogramms gestellt worden, wäre die chronisch-obstruktive TA in 19 % der Ohren diagnostiziert worden.

In der klinischen Routine wird das Auftreten von Cholesteatomen als Sekundärkomplikation und Indikator für eine Dysfunktion der TA aufgrund einer Gaumenspalte angesehen. Insgesamt wird angenommen, dass das Risiko der Cholesteatomentstehung bei Patienten mit Hart- und Weichgaumenspalten bis zu 200-mal höher ist als in der gesunden Bevölkerung [2, 39, 40]. Es ist nicht abschließend geklärt, ob diese Zahlen eine Folge der persistierenden Dysfunktion der TA sind oder ob die Erkrankungen des Mittelohrs eine Folge der unzureichenden Therapie im Kindesalter sind. Die Daten dieser Studie legen nahe, dass eine ausreichende Therapie von LKGS und chronisch-obstruktiver TA, z. B. mit Paukendrainagen, zu geringeren Raten von persistierender chronisch-obstruktiver TA sowie chronischen Mittelohrerkrankungen führen. In der Kohorte dieser Studie wurden signifikante Unterschiede zwischen den Gruppen für den semi-objektiven ETS-7-Score, aber nicht für den ETDQ‑7 gefunden, was die fehlende Symptomatik der chronisch-obstruktiven TA bei LKGS-Patienten unterstreicht. Obwohl signifikante Unterschiede zwischen den beiden Studiengruppen für den ETS-7-Score gefunden wurden, werden diese Unterschiede nicht als klinisch relevant angesehen, da die Medianwerte in beiden Gruppen nicht einer klinisch relevanten chronisch-obstruktiven TA entsprechen.

Für das Fehlen von Cholesteatomen oder Verwachsungen sowie den geringen Anteil an chronisch-obstruktiver TA in der Kohorte dieser Studie kommen mehrere Ursachen infrage. Zum einen könnte eine effektive Behandlung der Tubenfunktionsstörung in der Kindheit einen schützenden Effekt haben, zum anderen zielt die LKGS-Operation darauf ab, physiologische Bedingungen für die Sprach- und Essfunktionen zu schaffen, und kann folglich auch die Funktion der TA positiv beeinflussen. In einer Publikation zur Pathophysiologie der TA wurden insgesamt 15 LKGS-Patienten klinisch und mittels MRT untersucht. In 7 Fällen, bei denen sich eine chronische Mittelohrerkrankung zeigte, konnte eine deutliche Assoziation zu einer in der MRT nachgewiesenen Diskontinuität des M. tensor veli palatini nachweisen lassen [41]. Dies legt die Schlussfolgerung nahe, dass eine erfolgreiche chirurgische Behandlung die Entstehung chronischer Mittelohrentzündungen erheblich beeinflusst. Die in dem hier präsentierten Patientenkollektiv angewandte Technik nach Kriens [20] zeigt postoperativ bereits im Kindesalter funktionell günstige Ergebnisse hinsichtlich der velopharyngealen Kompetenz und der Tubenfunktion [42]. Die Ergebnisse der vorliegenden Studie zeigen weiterhin, dass die Rate der diagnostizierten chronisch-obstruktiven TA abnimmt, wenn mehr als eine Messung verwendet wird. Da die einzelnen Scores und Diagnoseinstrumente fehleranfällig sein und zu falsch-positiven Ergebnissen führen können, sollte die Diagnose von chronisch-obstruktiver TA nicht auf einer Methode allein beruhen. Bei Verwendung einer Kombination von pathologischen Scores in der ETS‑7 und dem ETDQ‑7 wurde die Diagnose einer obstruktiven TA nur in 6,3 % der Fälle gestellt.

## Limitationen

Diese Studie ist durch eine kleine Anzahl von Teilnehmern und eine ungleiche Größe der Studiengruppen begrenzt. Dennoch liegt die Stärke dieser Studie in der Untersuchung einer Kombination von subjektiven und objektiven Messungen zur Untersuchung der Funktion der TA bei Erwachsenen nach LKGS-Operation.

## Fazit für die Praxis

- Nach chirurgischer Behandlung der LKGS ist die Rate der persistierenden chronisch-obstruktiven TA bei Erwachsenen geringer als in früheren Studien berichtet.
- Eine sorgfältige Behandlung von Mittelohrpathologien und TA-Dysfunktionen im Kindesalter scheint erfolgreich zu sein.
- Für die Diagnostik wird eine Kombination aus objektiven, semi-objektiven und subjektiven Instrumenten empfohlen.
- Zudem werden regelmäßige ohrmikroskopische Untersuchungen des Trommelfells bei Patienten nach LKGS-Operation empfohlen.

## Supplementary Information





