# Supplementary material for: Eustachian tube dysfunction after cleft palate surgery: Use of the latest diagnostics
Source: HNO. 2022 Mar 22;70(7):557–63. [Article in German] doi: 10.1007/s00106-022-01147-z (PMC9242949; doi:10.1007/s00106-022-01147-z)
Supplement: Supplementary file 1 [file 106_2022_1147_MOESM1_ESM.pdf]

Universitätsmedizin Rostock

Dokumentationsbogen
HNO-Klinik *Tubendilatation*

Patient:

Geschlecht: Alter:

Datum der Untersuchung:

		ETS-7	
Seite		**re**	**li**
**Tuben-öffnung beim Schlucken**	Ja (2 P.)		
	ab&zu (1 P.)		
	Nein (0 P.)		
**Valsalva positiv**	Ja (2 P.)		
	ab&zu (1 P.)		
	Nein (0 P.)		
**TMM 30** mbar R-Wert (R ≤ 1: 2 P.; R > 1: 1 P.; kein R: 0 P.)			
**TMM 40** mbar R-Wert (R ≤ 1: 2 P.; R > 1: 1 P.; kein R: 0 P.)			
**TMM 50** mbar R-Wert (R ≤ 1: 2 P.; R > 1: 1 P.; kein R: 0 P.)			
**Objektiver Valsalva** (Punkte für Positiv=2, schwach/verzögert=1, negativ=0)			
**Tympanogramm nach Jerger** (Punkte für Typ A=2, Typ C=1, Typ B=0)			
**Score gesamt**			
