# Supplementary material for: Eustachian tube dysfunction after cleft palate surgery: Use of the latest diagnostics
Source: HNO. 2022 Mar 22;70(7):557–63. [Article in German] doi: 10.1007/s00106-022-01147-z (PMC9242949; doi:10.1007/s00106-022-01147-z)
Supplement: Supplementary file 2 [file 106_2022_1147_MOESM2_ESM.pdf]

HNO-Klinik

ETDQ-7
Anamnesefragebogen

Universitätsmedizin Rostock

Patient:

Geschlecht: Alter:

Datum:

Liegen folgende Beschwerden/Befunde bei Ihnen vor?

- Druckgefühl oder Schmerzen im Ohr z.B. bei Berg- und Talfahrt: □ rechts □ links
- Verschwinden des Druckgefühls durch Schlucken: □ rechts □ links
- Knacken im Ohr beim Schlucken: □ rechts □ links
- Druckausgleich durch Valsalva: □ rechts □ links
- Häufige oder chronische Mittelohrentzündungen: □ rechts □ links
- Ohrenlaufen: □ rechts □ links
- zurückliegende Ohroperationen: (bitte hier notieren)

Wie sehr haben Sie folgende Probleme im letzten Monat beeinträchtigt?

Beschwerden	kein Problem		mäßiges Problem			schweres Problem	
**1. Druckgefühl im Ohr**	**1**	**2**	**3**	**4**	**5**	**6**	**7**
**2. Schmerzen in den Ohren**	**1**	**2**	**3**	**4**	**5**	**6**	**7**
**3. Das Gefühl die Ohren seien verstopft oder „unter Wasser“**	**1**	**2**	**3**	**4**	**5**	**6**	**7**
**4. Ohrbeschwerden im Rahmen von einfachen Erkältungen und Nebenhöhlenentzündungen**	**1**	**2**	**3**	**4**	**5**	**6**	**7**
**5. Knisternde oder Knallende Geräusche in den Ohren**	**1**	**2**	**3**	**4**	**5**	**6**	**7**
**6. Klingeln in den Ohren**	**1**	**2**	**3**	**4**	**5**	**6**	**7**
**7. Das Gefühl das Hören sei dumpf**	**1**	**2**	**3**	**4**	**5**	**6**	**7**

Gesamtpunktzahl: ____________________
